# WeMo: A Prototype of a Wearable Mobility Device Adapting to User’s Natural Posture Changes

**DOI:** 10.3390/s23187683

**Published:** 2023-09-06

**Authors:** Yang Chen, Takashi Kuwahara, Yuki Nishimura, Kenji Suzuki

**Affiliations:** 1 Artificial Intelligence Laboratory, Institute of Systems and Information Engineering, University of Tsukuba, Tsukuba 305-8577, Japan; chenyang@ai.iit.tsukuba.ac.jp; 2 Ph.D. Program in Empowerment Informatics, School of Integrative and Global Majors, University of Tsukuba, Tsukuba 305-8577, Japan; kuwahara@golem.iit.tsukuba.ac.jp; 3 Research Center for Intelligent Robotics, Research Institute of Interdisciplinary Innovation, Zhejiang Lab, Hangzhou 311121, China; 4 Center for Cybernics Research, Institute of Systems and Information Engineering, University of Tsukuba, Tsukuba 305-8577, Japan; kenji@ieee.org

**Keywords:** personal mobility device, wearable mobility, micro mobility device, smart mobility device

## Abstract

Mobility is fundamental for human beings. In the current society, many personal mobility solutions have been invented to enable more time-efficient mobility, such as self-balancing vehicles, electric unicycles, and electric scooters. Personal mobility devices can provide flexibility to transportation. However, most personal mobility devices need to be carried by their users in the case that they climb stairs and steps. Therefore, many researchers have focused on developing stair-climbing vehicles, but due to the complicated mechanism, these devices are usually huge and heavy. To realize a new type of personal mobility device with more flexibility, we proposed a novel concept of a personal mobility device design that combines the agile mobility of a wheel type mechanism but does not limit a human’s natural stair climbing ability. In this study, we introduced a compact personal mobility device, namely WeMo, under the concept of “wearing mobility”, which extends humans’ mobility in daily life. The developed hardware realizes “walking mode” and “driving mode”. Users can move with the motorized driven wheels of the device during driving mode, and users can walk on their feet without any interference from the device during walking mode. In this manuscript, the detailed design of the hardware and control strategy were explained first.Then, we conducted fundamental user tests and discussed the ability of the developed device from test results. Finally, the conclusions and future work were provided.

## 1. Introduction

A smart city is defined as a city in which Information and Communications Technology (ICT) is merged with traditional infrastructures, coordinated and integrated using new digital technologies [1]. In a smart city, new types of transportation are being explored to ensure greater and more effective mobility. Mobility is fundamental for human beings. Many personal mobility device solutions have been invented and introduced to enable more time-efficient and environment-friendly transportation for the current society. A personal mobility device, also known as smart mobility or micro mobility, is described as any assistive device that facilitates individual human transportation [2]. Various product types of personal mobility devices are available; for example, electric unicycles, kick electric scooters, electric scooters, three-wheeler electric scooters, electric mobility carts, electric bicycles, hoverboards, Segway, and electric caster boards were listed in a survey study of personal mobility devices [3]. Personal mobility devices can provide flexibility to their users while covering the first/last mile of a multi-modal trip [4]. However, most personal mobility devices have a disadvantage in the case of climbing stairs and steps. The users of current personal mobility devices must carry their devices with their hands when moving on stairs and steps. The well-known Segway [5] is a compact solution for a personal mobility device, and Segway has been the most common device type compared with other types of device [6]. However, the weight of the device is 12.8 kg, even for its lightest version. This is still a large burden in case the user needs to carry it using their hands to climb stairs. Similarly, Sasaki et al. developed a personal riding-type wheeled mobile platform, and its weight was 12 kg [7,8].

To solve the problem of stairs, many researchers have focused on developing stair-climbing vehicles, such as [9,10,11,12]. Podobnik et al. developed an electronic wheelchair with a hybrid mechanism of wheels and tracked wheels as their entry into the Cybathlon competition, which promotes the development of advanced robotic devices for people with disabilities [9]. Sugahara et al. developed a wheelchair with transformable wheeled four-bar linkages to climb stairs in historical sites [10]. Maeda et al. developed a wheelchair that has wheel mode for moving by wheels on a flat surface and leg mode for overcoming stairs [11]. However, due to the complicated mechanism, these devices are usually huge and heavy. The hybrid-type wheelchair by Podobnik et al. is 160 kg [9], the wheelchair with linkages by Sugahara et al. is 154 kg [10], and the wheelchair that has leg mode by Maeda et al. is 92 kg [11]. Other than electric wheelchairs, devices that disabled and elderly persons can ride on in a standing position have been proposed in previous studies [13,14]. These personal mobility devices showed significant improvement in user mobility and were widely accepted by users. However, these devices also have problems with huge weight (<100 kg [13]) and climbing stairs. Exoskeletons are another category that enables the user to have mobility on both flat surfaces and stairs. Most of them are developed for lower body impaired people, such as [15,16]. Other works have also been done to enhance the mobility of healthy people such as an ultra-lightweight wearable robot in [17] and a soft exo-suit introduced in [18]. However, as a sacrifice, when moving on flat surfaces, bipedal locomotion is less efficient than wheeled-type locomotion.

Considering situations when users carry or store the device, reducing device weight is key to removing the pain of using personal mobility devices. In addition, the above-described electric wheelchairs have been developed as assistive devices for empowering disabled people through robotic technologies, as represented by the study by Morbid et al. [12]. In this study, we diverted those concepts and technologies to personal mobility devices for general users. The novelty lies in the concept of “a personal mobility device that can be worn” and its design of a proposed wearable mobility device that combines the agile mobility of wheel-type mechanisms but does not limit a human’s natural posture change. In our previous project, we presented a wearable mobility device in [19]. However, when using this device, the user needed to equip the main device and also additional wheels on their foot, which is inconvenient for wearing and walking. In addition, the methodology of developing the hardware and control was not well considered.

**Figure 1:**
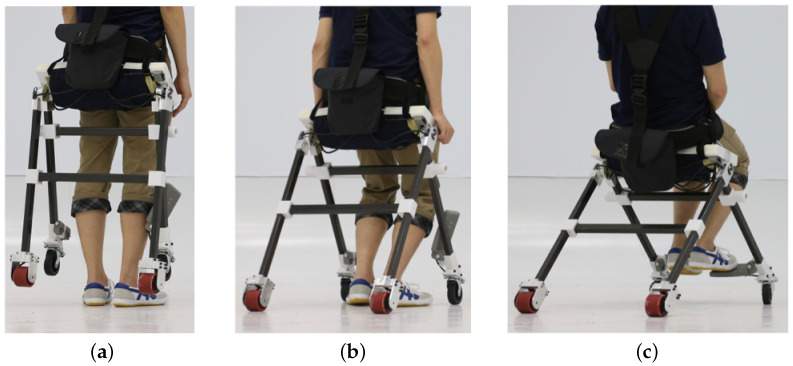
Conceptual image of WeMo. (**a**) Standing posture. (**b**) Transformation adapting to user’s natural posture change. (**c**) Sitting posture.

In this study, we introduced a new type of compact personal mobility device, namely “WeMo”, under the concept of “wearing mobility”. Our new prototype adapts to the user’s natural posture change. “Natural posture” in this manuscript means a human’s natural standing-to-sitting and sitting-to-standing motion and shifting body posture when trying to turn. Our device adapts to the user’s natural posture because when the user stands up, the device passively transforms into standing mode without extra effort from the user, as well as when the user sits down. When turning around, the control algorithm is based on the user’s natural body shifting, which agrees with the finding introduced in [20] that the body’s center of mass shifts and the trunk rolls toward the inner side of the turning during a human’s natural walking. We developed the device to extend human mobility in daily life. The proposed design enables faster wearability and higher stability. A conceptual image of the proposed device is shown in Figure 1. In this manuscript, we conduct the following design practices and tests to realize the proposed device. This manuscript shows the following:

(1) The device hardware design, which transforms according to the user’s natural posture change.
(2) The user interface, which controls the device according to body movements.
(3) The ability of the device in situations of daily life, including stair climbing.

The detailed design of the hardware and control strategy is explained in Section 2, and fundamental user tests are conducted in Section 3. We discuss the ability of the developed device from test results in Section 4. Finally, the conclusions and future work are provided in Section 5.

## 2. Methodology

To realize the proposed concept of “wearing mobility”, we developed a novel mobility device in this study. The developed hardware realized “walking mode” and “driving mode”. Users can move with the motorized driven wheels of the device during driving mode, and users can walk on their feet without any interference from the device during walking mode. The frame of the developed wearable device was designed not to disturb users’ walking process during walking mode. The developed device has two kinds of sensors: pressure sensors and a load cell. These sensors were used to control the direction and speed of the device during driving mode. In the following subsections, hardware design, sensing, and control units are described in detail.

**Figure 2:**
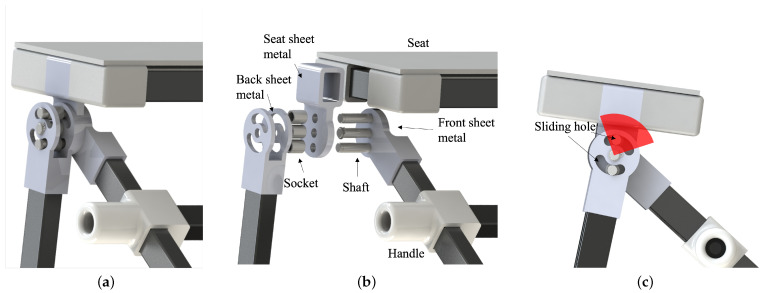
Joint view. (**a**) Joint. (**b**) Joint exploded view. (**c**) An inter-medium status of the rotation. The red area indicates the rotation range.

**Figure 3:**
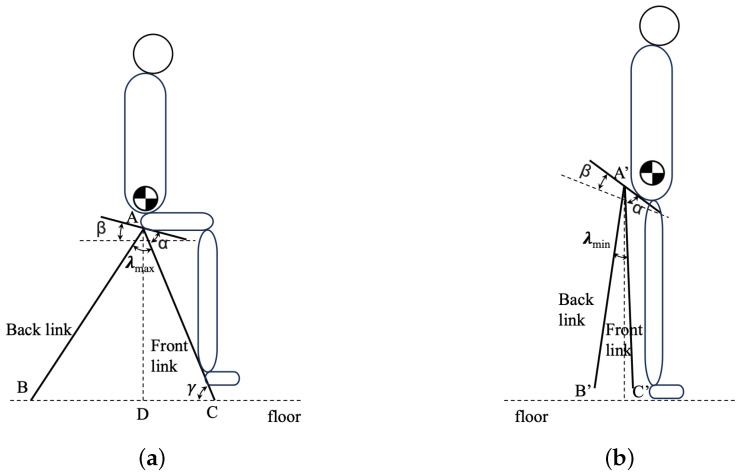
Simplified model of WeMo. (**a**) In sitting posture. (**b**) In standing posture. AB and AC represent the back and front links, respectively. *A* denotes the revolute joint of AB, AC, and the seat. AD is perpendicular to the floor. $A^{\prime }$, $B^{\prime }$, $C^{\prime }$ are corresponding points of *A*, *B*, *C* in standing posture.

The developed device can realize two states of formation: “driving mode” and “walking mode”. In the proposed design, to realize the smooth transformation of the device frame, we developed a particular joint made with an aluminum alloy, as shown in Figure 2. The seat sheet metal is connected with the front sheet metal and back sheet metal through three shafts. The seat sheet metal is fixed stably with the front one, while the back one is only fixed stably in the extension axis. The rotation range is regulated by the space, as shown in Figure 2c. The purposes of this design are to enable (1) high wearability in standing posture, (2) stable support in sitting posture, and (3) smooth transformation between two postures. A simplified model of WeMo in sitting and standing postures is shown in Figure 3. The length between the hip joint and the ground is different for individuals. However, considering the height of most chairs is from 0.43 m to 0.56 m [21], AD was selected as 0.48 m in the current design. Considering the ratio between the thigh and the lower leg and easiness for the user to press the foot pedal, we set the angle γ as 60°; therefore, AC equals 0.55 m. The length of BD was selected to provide stable support when the human is in a sitting posture, while AB connects *A* through a revolute joint, which means that it rotates freely between sitting and standing postures. However, AB is constrained under the condition that it should not touch the ground when the user is in a standing posture, altogether resulting in AB being 0.67 m, which was calculated by Equation (1).

(1) $$AB=\sqrt{AD^{2}+BD^{2}}.$$

where AB is the length of the back link, AD is the length between the joint and the ground, and BD is the distance between the perpendicular line extending from the joint to the ground and the ground contact point of the back link. Therefore, the maximum angle of BAC can be calculated by Equation (2):

(2) $$\lambda_{max}=\arctan\left(\frac{BD}{AD}\right)+\frac{\pi }{2}-\gamma .$$

where $\lambda_{max}$ is the maximum angle of two links, and γ is the angle of the front link and the ground. β was designed to be 25° to provide a comfortable sitting forward moving posture. Therefore, α=γ−β equals 35°. With a suitable harness design, the user is able to move into a standing posture easily, and with a minimum angle of $B^{\prime }A^{\prime }C^{\prime }$ of $\lambda_{min}$, the link of $A^{\prime }B^{\prime }$ and $A^{\prime }C^{\prime }$ would be on the left and right sides of the vertical line, making it easy for the user to transform back into a sitting posture.

**Figure 4:**
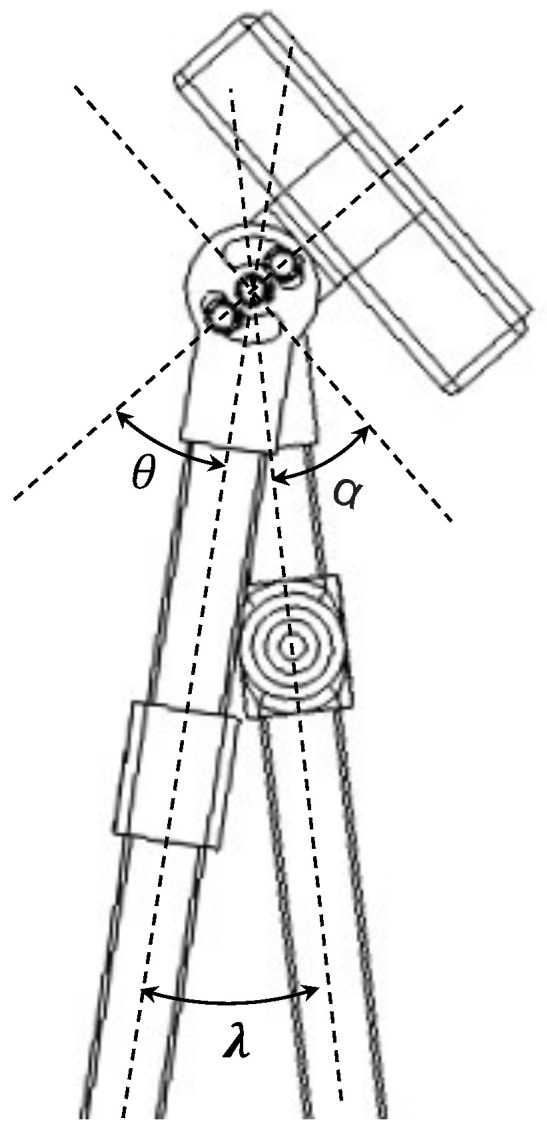
The relation between θ and λ. θ is the angle from AB to the connecting line (as well as the centerline of the seat) of the three shafts depicted in Figure 2c. The length of the sliding hole is decided by the left and right limits of θ.

We realize the required $\lambda_{min}$ and $\lambda_{max}$ by setting up the left and right limit of the sliding hole, which are calculated as $\theta_{left}$ and $\theta_{right}$ in Equation (3). As shown in Figure 4, θ is the angle from AB to the connecting line of three shafts, with a clockwise direction as positive. The left limit position of the sliding hole blocks the link AB to continue rotating counter-clockwise in standing posture, which corresponds to $\lambda_{min}$. The right limit position of the sliding hole blocks the link AB to continue rotating clockwise in sitting posture, which corresponds to $\lambda_{max}$. The range of the sliding hole $\theta_{range}$ is then obtained.

(3) $$\left\{\begin{array}{c} \theta =\frac{\pi }{2}-\alpha -\lambda \\ \theta_{left}=\frac{\pi }{2}-\alpha -\lambda_{min}\\ \theta_{right}=\frac{\pi }{2}-\alpha -\lambda_{max}\\ \theta_{range}=\lambda_{max}-\lambda_{min} \end{array}\right.$$

### 2.1. Hardware Design

#### 2.1.1. Driving Mode

**Figure 5:**
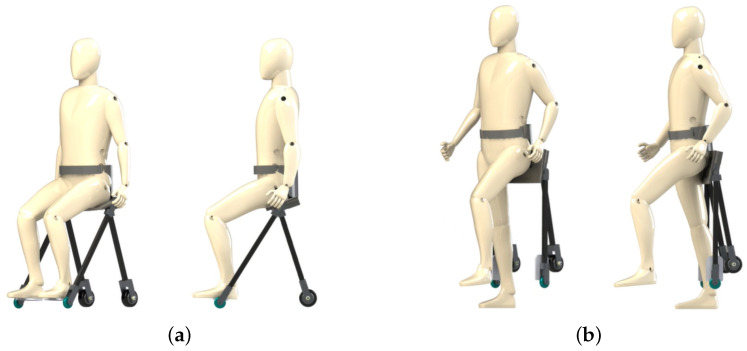
Two modes of the proposed mobility device. (**a**) Driving mode. (**b**) Walking mode.

“Driving mode” is the state in which users move with the motorized driven wheels of the device (see Figure 5a). During driving mode, the stability of the device is one of the important factors. When the position of the seat is placed too high from the ground, the device will be unstable because of the high position of the center of gravity. On the other hand, when the position of the seat is placed too low, users cannot stand up once they sit down on the device for driving mode. Therefore, in our design, we used two free-rotation wheels on the front and two driving wheels on the back. The four contact points to the ground make the device stable while driving. Stability is an important factor for ease of use. As described in Azizi et al. [22], an unstable device such as a self-balanced device is difficult to control for some users [22].

#### 2.1.2. Walking Mode

“Walking mode” is the state in which users walk on their feet without any interference from the device (see Figure 5b). Since the device cannot generate any support for users during walking mode, the device was designed not to disturb users’ walking motion. The width between both sides of frames is widened compared to the previous design [19] because the frame touches users’ legs when the width of the side frames is narrow. In addition, the device was made using Carbon Fiber Reinforced Plastics (CFRP) and aluminum alloy. This was done to reduce the weight of the device and make it a lightweight device for easy wearing. Moreover, in the actual design of the developed device, we implemented a shoulder belt as well as a waist belt. During walking mode, it is difficult to hold the device steady only with the waist belt, and the weight of the device can be a burden for users’ backs. By installing shoulder belts, this burden could be redistributed.

### 2.2. Sensing and Controlling

**Figure 6:**
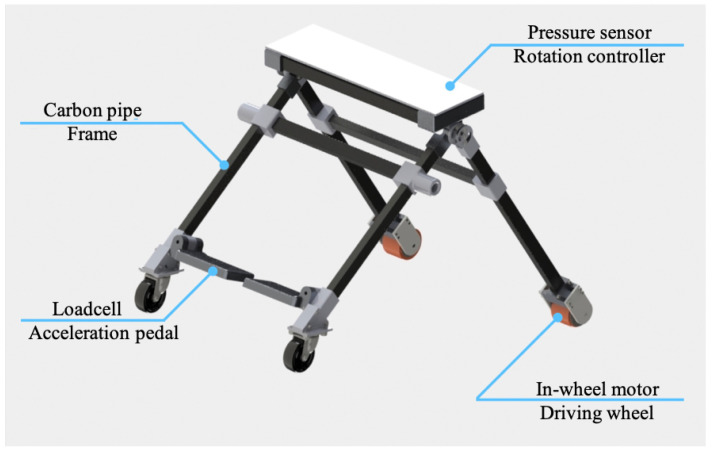
Computer-aided design of the proposed device.

**Figure 7:**
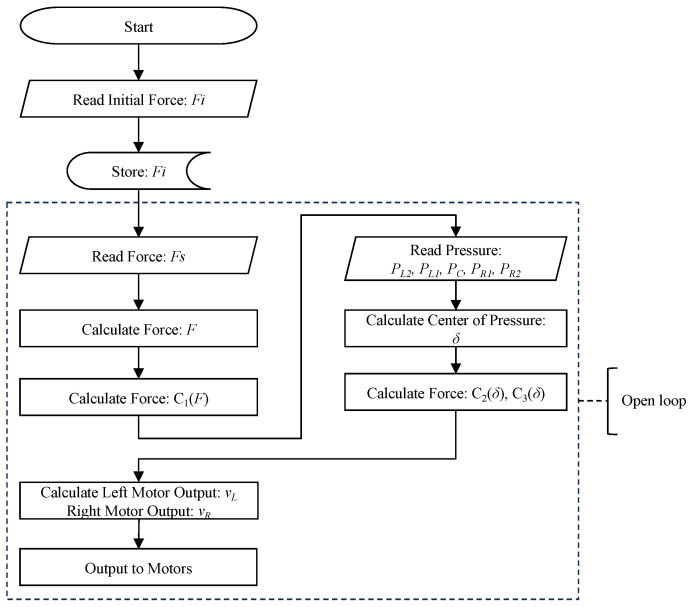
Flow of speed and direction control.

During driving mode, users need to control the direction of movement and speed of driving. As depicted in Equation (4), *v* and *w* denote linear and angular velocity, respectively. $C_{1}$ controls the velocity magnitude, while $C_{2}$ and $C_{3}$ control the velocity direction by adjusting the relative weight between *v* and *w*. In the proposed device, we realized direction and speed control by two sensing units: a pressure sensor unit and a load cell sensor unit. As shown in Figure 6, the pressure sensor unit was installed on a seat of the device, and the load cell sensor unit was installed on an acceleration pedal of the proposed device’s frame.

(4) $$\left\{\begin{array}{c} v\left(F,\delta \right)=C_{1}\left(F\right)C_{2}\left(\delta \right)\\ w\left(F,\delta \right)=C_{1}\left(F\right)C_{3}\left(\delta \right) \end{array}\right.,$$

where v(F,δ) and w(F,δ) are determined by force reading from the acceleration pedal *F* and the center of pressure δ. $C_{1}\left(F\right)$ is the function to control the velocity of the device according to *F*. $C_{2}\left(\delta \right)$ and $C_{3}\left(\delta \right)$ are to control the direction by δ. According to the kinematics of the robot, the velocity of the left and right wheels is converted as Equation (5). Then, a low-level controller converts the velocity to Pulse Width Modulation (PWM), which is the output to the motor with a wheel of its radius of *R*.

(5) $$\left\{\begin{array}{c} v_{L}=v-Rw\\ v_{R}=v+Rw \end{array}\right.$$

where $v_{L}$ and $v_{R}$ are the velocities of the left and right side, respectively. The general control flow is shown in Figure 7. The details are explained in Section 2.2.1 and Section 2.2.2.

#### 2.2.1. Pressure Sensors and Direction Control

The method of locomotion direction control based on pressure sensing was presented in the previous study [13]; however, the sensing surface is installed in front of the user’s waist level, while in this study, the sensing surface is on the seat, which leads to a difference in the mapping from the body motion to robot motion. The design principle is as follows: when the user shifts the body to one side, the device turns to the corresponding side, which agrees with the finding introduced in [20] that the body’s center of mass shifts and the trunk rolls toward the inner side of the turning during a human’s natural walking.

**Figure 8:**
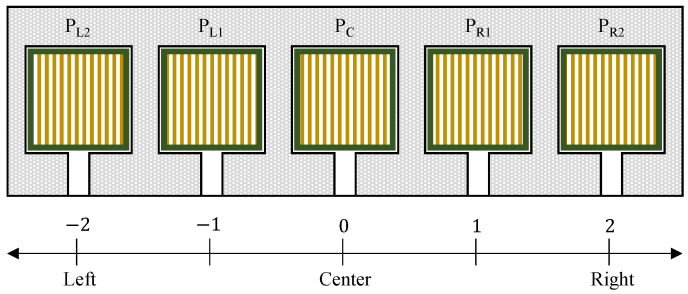
Array of pressure sensors on the seat.

Five pressure sensors (FSR406) were attached to the seat of the device. First, we measured the pressure values of five sensors as $P_{L2}$ on the outer left, $P_{L2}$ on the inner left, $P_{C}$ on the center, $P_{R1}$ on the inner right, and $P_{R2}$ on the outer right of the seating area (see Figure 8). With $P_{L2}$ to $P_{R2}$, the center of pressure (δ) was calculated by using Equation (6).

(6) $$\delta =\frac{-2P_{L2}-P_{L1}+P_{R1}+2P_{R2}}{2\left(P_{L2}+P_{L1}+P_{C}+P_{R1}+P_{R2}\right)}$$

**Figure 9:**
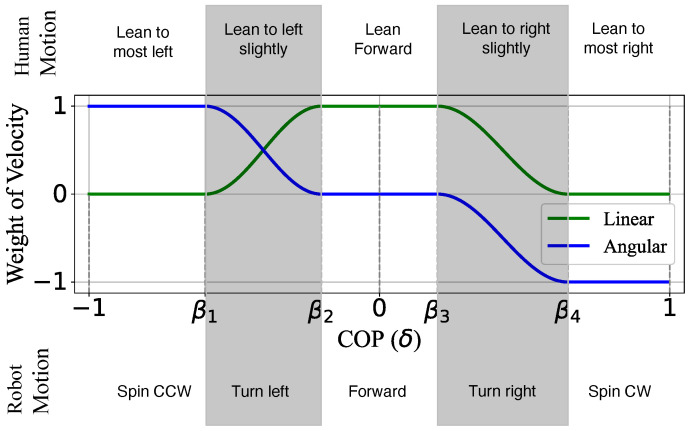
The mapping from human motion to velocity weight and robot motion.

With Equation (6) δ is normalized to [−1,1]. According to δ, the direction of driving was decided by the weight distribution of linear and angular velocity, which are calculated in $C_{2}\left(\delta \right)$ and $C_{3}\left(\delta \right)$, as shown in Equations (7) and (8).

(7) $$C_{2}\left(\delta \right)=\left\{\begin{array}{cc} 0, & -1\leq \delta <\beta_{1}\\ \frac{1}{2}+\frac{1}{2}\sin\left(\frac{\pi }{\beta_{1}-\beta_{2}}\left(\delta -\beta_{1}\right)+\frac{\pi }{2}\right), & \beta_{1}\leq \delta <\beta_{2}\\ 1, & \beta_{2}\leq \delta <\beta_{3}\\ \frac{1}{2}+\frac{1}{2}\sin\left(\frac{\pi }{\beta_{4}-\beta_{3}}\left(\delta -\beta_{3}\right)+\frac{\pi }{2}\right), & \beta_{3}\leq \delta <\beta_{4}\\ 0, & \beta_{4}\leq \delta <1 \end{array}\right.$$

(8) $$C_{3}\left(\delta \right)=\left\{\begin{array}{cc} 1, & -1\leq \delta <\beta_{1}\\ \frac{1}{2}+\frac{1}{2}\sin\left(\frac{\pi }{\beta_{1}-\beta_{2}}\left(\delta -\beta_{1}\right)+\frac{\pi }{2}\right), & \beta_{1}\leq \delta <\beta_{2}\\ 0, & \beta_{2}\leq \delta <\beta_{3}\\ -\frac{1}{2}-\frac{1}{2}\sin\left(\frac{\pi }{\beta_{4}-\beta_{3}}\left(\delta -\beta_{4}\right)+\frac{\pi }{2}\right), & \beta_{3}\leq \delta <\beta_{4}\\ -1, & \beta_{4}\leq \delta <1 \end{array}\right.$$

where $\beta_{1}$ to $\beta_{4}$ are classification points for distinguishing different body postures depending on the location of δ. With this, we expect to realize a natural mapping from human motion to velocity and robot motion as shown in Figure 9. For example, when the user shifts his/her body slightly to the right side, the value of $P_{R1}$ and $P_{R2}$ increases as well as δ; then, δ is located between $\beta_{3}$ and $\beta_{4}$, the weight of linear and angular velocity decreases to [0,1] and [−1,0] individually. A negative value means clockwise turning for angular velocity. Therefore, the device drives to the right.

#### 2.2.2. Load Cell and Speed Controlling

The speed of the robot was controlled by an acceleration pedal with a load cell. The pedal was installed on the front-right frame of the developed device. The main body of the pedal was made by a 3D printer. Underneath the pedal, the load cell (single-point load cell with a range of 0–20 kg) was installed to measure the pressing force of the user’s foot. In the beginning, the initial pressing force when the user simply places his/her foot on the pedal was measured as $F_{i}$. This initial force is always deducted from further sensor readings ($F_{s}$) to avoid changing speed due to the individual’s leg weight. The force sensor value *F* is calculated as shown in Equation (9).

(9) $$F=F_{s}-F_{i}$$

Then, Equation (10) is used to calculate the velocity magnitude $C_{1}\left(F\right)$.

(10) $$C_{1}\left(F\right)=\left\{\begin{array}{cc} 0 & F\leq 0.2F_{m}\\ k_{1} & 0.2F_{m}<F\leq 0.4F_{m}\\ k_{2} & 0.4F_{m}<F\leq 0.6F_{m}\\ k_{3} & 0.6F_{m}<F\leq 0.8F_{m}\\ k_{\max} & 0.8F_{m}<F\leq F_{m} \end{array}\right.$$

where $F_{m}$ represents a maximum load cell sensor value. $k_{1}$ to $k_{\max}$ represent speed magnitude levels ($0<k_{1}<k_{2}<k_{3}<k_{max}$).

### 2.3. Developed Wearable Mobility Device

**Figure 10:**
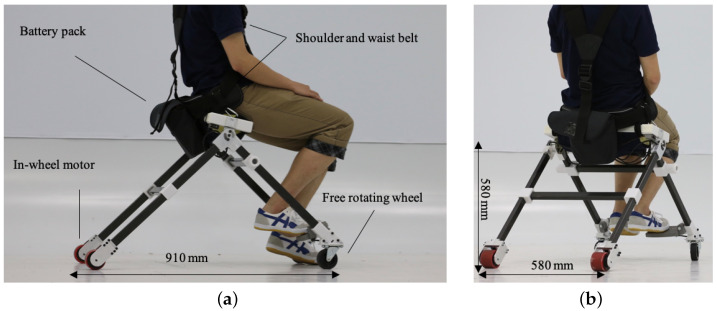
Developed device in sitting posture. (**a**) Side view. (**b**) Back left view.

**Figure 11:**
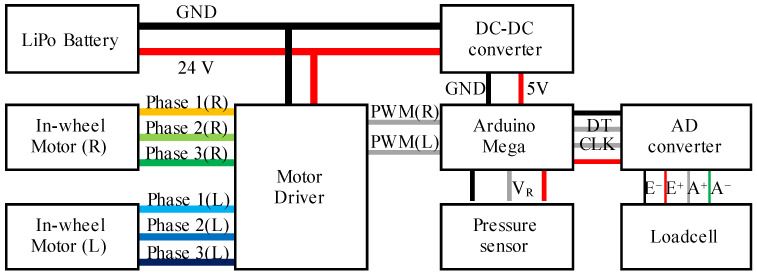
Overview of the electronic circuit.

Figure 10 shows an overview of the developed wearable mobility device. The device mainly consists of CFRP frames, 3D printed joint parts, two free-rotation casters on each end of front frames, two in-wheel driving motors, and waist/shoulder belts. The joint parts were made of an aluminum alloy. The sensing unit consists of pressure sensors inside the seat cushion and the load cell on the pedal. We used Arduino Mega as the control unit. Two in-wheel motors were controlled by motor drivers individually. An overview of the electronic diagram is shown in Figure 11. The sizes of the developed device in sitting posture were 580 mm, 910 mm, and 580 mm, while the sizes of it in standing posture were 760 mm, 210 mm, and 580 mm. The total weight including a battery was 7.9 kg. The battery was stored in a battery pack behind the user’s back.

## 3. Experiments

In this paper, we conducted the following experiments to confirm the capability of the proposed design as a wearable mobility device. The experiment was separated into four parts: frame transformation test, mobility test, walking and climbing test, and control test. We demonstrated that the user can walk while wearing our device and that the device can carry the user to the destination. In addition, we demonstrated direction and speed control tests.

### 3.1. Frame Transformation Test

**Figure 12:**
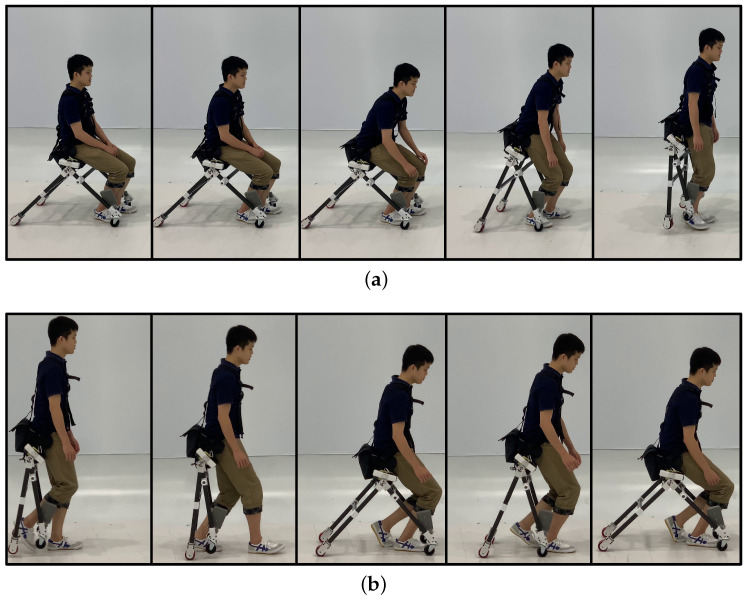
Standing and sitting motion sequences. The duration between frames was 0.5 s. (**a**) Standing up motion. (**b**) Sitting down motion.

In the proposed concept, users can easily change their status from driving to walking. In this subsection, a user stands up and sits down while wearing the developed wearable mobility device. Figure 12a shows the process of standing up movement. As shown in the figure, the frames were automatically bent so as not to disturb walking. Figure 12b shows the process of sitting down while wearing the device. The frames opened once the wheels touched the ground and let the user sit down on the stable formation of the device.

### 3.2. Control Test

**Figure 13:**
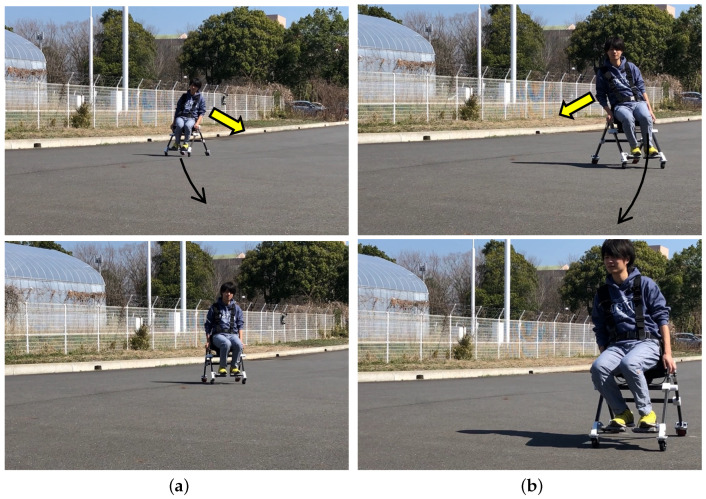
Control experiment: the yellow arrow indicates the direction where the user shifted the body, and the black arrow indicates the moving direction of the device. The observed device speed was 14.5 km/h. (**a**) Left turn. (**b**) Right turn.

We conducted the fundamental test to confirm the controlling method using the center of pressure measured by the developed sensing unit. As a result, we could observe that when the user tilted his body to one side, the device moved to that side. As shown in Figure 13a, when the user leaned his body to the left in the direction of the yellow arrow, the device turned left. Conversely, as shown in Figure 13b, when the user leaned to the right, we observed that the travel direction changed to the right. The travel direction is indicated by black arrows. The observed device speed was 14.5 km/h when the device was under control in straight-line movement.

### 3.3. Walking and Climbing Test

**Figure 14:**
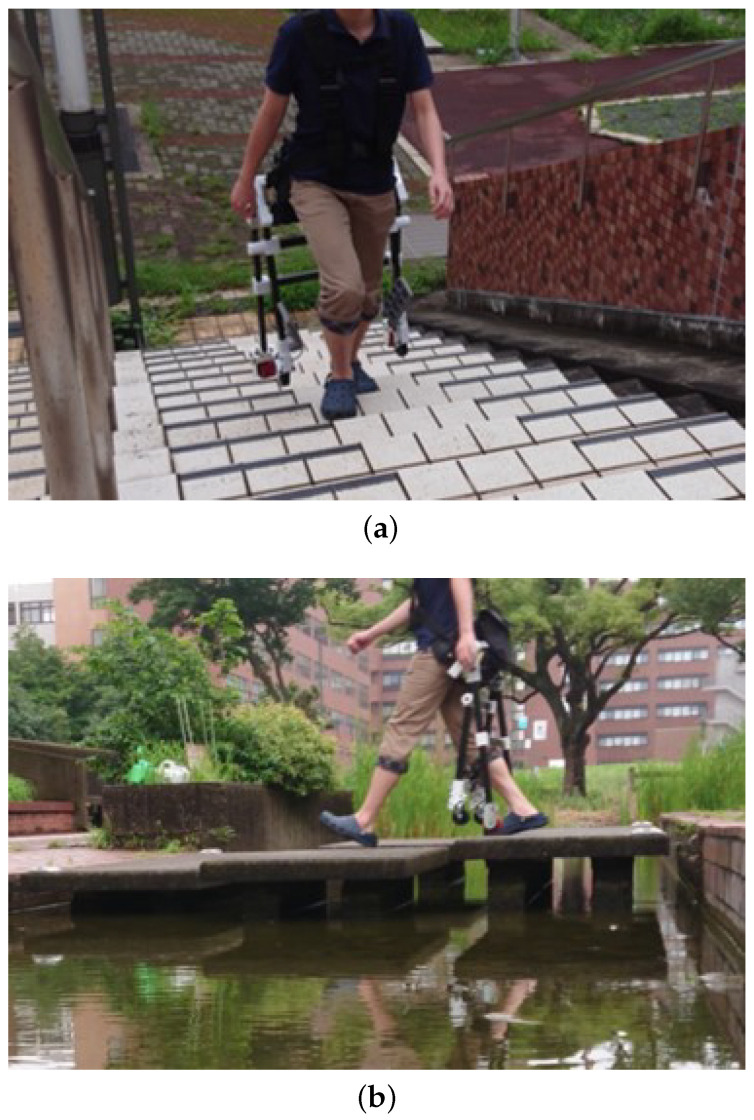
Walking and climbing test. (**a**) Climbing up stairs. The stair angle was 28°. (**b**) Walking on pedestrian bridge. The gaps were 120 to 130 mm.

The frame transformation test showed that the device frame did not interfere with the user’s motion in standing position (see Figure 12a). Therefore, we determined that the frame did not interfere with users’ walking and stair-climbing motion as well. One of the strong and original points of wearable mobility devices is that users can move on their feet and climb when they are facing stairs or steps. Figure 14a shows the result of the stair climbing test. The user could use stairs while the frame did not touch either the user’s body or the ground. Figure 14b shows the result of moving on a bridge that was constructed only for pedestrians because there are gaps on the bridge. In case users use other wheeled-type mobility devices, they must carry the device in their hands when moving in these places. However, our device can easily change driving mode to walking mode to climb stairs and cross the bridge.

### 3.4. Mobility Test

**Figure 15:**
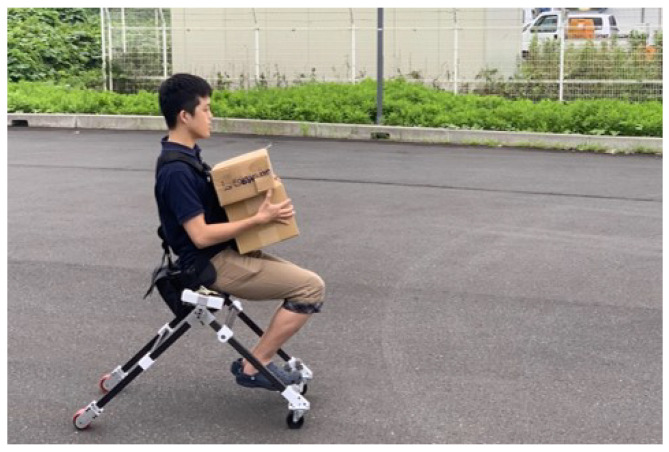
Driving experiment with a user carrying boxes.

**Figure 16:**
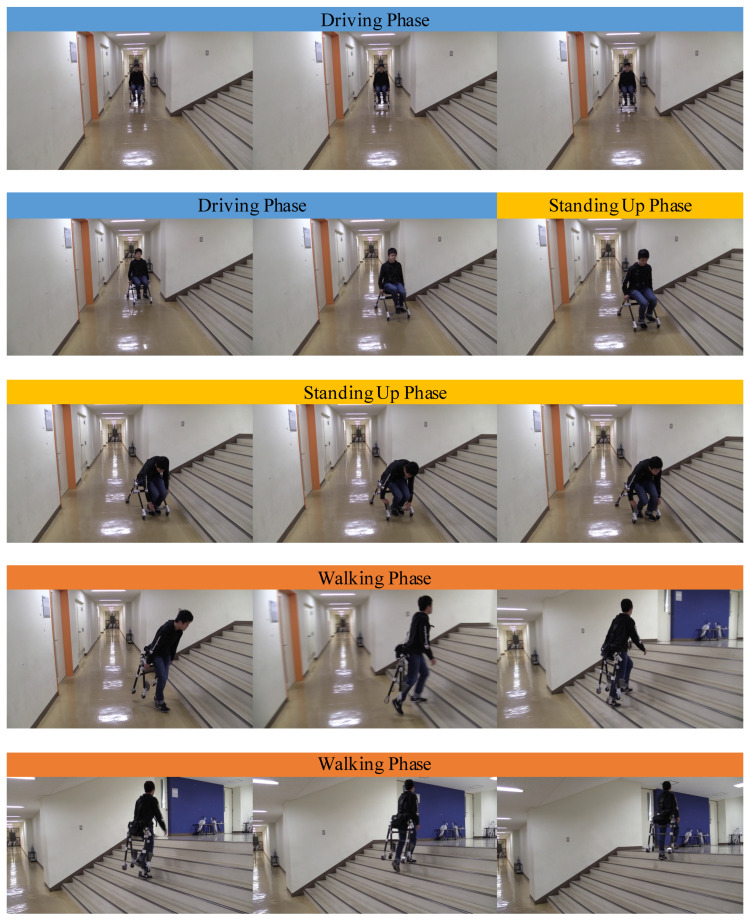
Mobility experiment in daily life setting. The stair angle was 34°.

Figure 15 shows a scene of the user carrying boxes while driving the proposed device. Since our design does not require users to carry the device with their hand when facing stairs, the user of our device can continue to carry the boxes. The driving mode and walking mode of the device were tested on flat surfaces and stairs in real life. As shown in Figure 16, we could observe that users drove the device on a flat floor and then climbed the stairs.

## 4. Discussion

First, in the frame transformation test, we confirmed that the device deformed according to the user’s natural movements of standing and sitting. As described in the hardware design, the joints functioned correctly. The frames were opened and closed when the user sat down and stood up. The device speed observed in Figure 13 was 14.5 km/h, while the maximum speed of electric wheelchairs is 6 km/h. Therefore, we need to consider setting a limitation of the device speed up to 6 km/h to ensure safety. The maximum stair-climbing rate was around 34°, which was observed from the experiment shown in Figure 16. Regarding the standard choice for the control method of personal mobility devices, electric scooters use a handlebar to control the device, and electric wheelchairs use a joystick. These control methods are simple but keep the user’s hands on the control. We proposed the control method using the body’s center of pressure. The advantage of the proposed control system lies in the hands-free feature, which is suitable for the compact design of the proposed device. In the following control test, we confirmed that it is possible to control the device using a sensor unit consisting of pressure sensors and a load cell when the user wears the device in driving mode. However, in this experiment, we did not record the reading value of each sensor and the actual output value because the current device does not have a data-logging function. A data logger and motion capture system should be implemented to record sensor input and device output to evaluate the usability of the device in the future. The stair climbing test proved the original concept of the proposed design, which enables users to overcome stairs and steps. The final mobility test shows the potential of devices to improve mobility in the situations we encounter in our daily lives. However, this test allowed us to discover an improvement point of the device. As shown in the middle part of Figure 16, the acceleration pedal had to be manually held back when the user stood up. It will be necessary to have a mechanism that detects the user’s standing-up and sitting-down motion and then holds the pedal back and opens the pedal automatically.

### Limitation

The driving mode of the current device mainly targets the flat surface, which is more suitable for the urban environment. The limitation on its stability is that four wheels must be touched to the ground at the same time while driving to ensure stability. The limitation of the versatility of the current device is that it can only manage flat surfaces. The target scenario would be extended to uneven terrains with a greater size of wheels and suspension mechanism. In this manuscript, the safety and reliability were not evaluated. Due to the limitations on safety and reliability, the device cannot be used on public roads in its current form. To apply it to the public, administrative approval is necessary. Further development of the wearable mobility device to suit policy and regulation is required for use on public roads [23].

## 5. Conclusions and Future Work

In this study, a novel prototype of a personal mobility device was designed to be used in a smart city. The developed device can be merged with traditional infrastructures. The device showed the possibility to ensure greater and more effective mobility. In this manuscript, we proposed and developed a novel solution for personal transportation based on the concept of “a personal mobility device that can be worn”. The hardware design and control algorithm are formalized and implemented. The developed device has made it possible to reduce the burden of carrying the device by hand and use it even in a place where there are obstacles such as steps and stairs. We conducted four types of tests in Section 3. Although our experiment consisted of only fundamental qualitative tests, we have shown the novelty of the device.

In future work, it will be necessary to quantitatively evaluate and set parameters such as optimal thresholds of sensors. In addition, user studies on satisfaction and safety should be conducted for comparison with other types of devices [24].

## Data Availability

The supplemental video is provided on YouTube at https://www.youtube.com/watch?v=PAKdDLtD624, accessed on 9 August 2023.

## Abbreviations

The following abbreviations are used in this manuscript:

ICT	Information and Communications Technology
WeMo	Wearable mobility
CFRP	Carbon Fiber Reinforced Plastics
PWM	Pulse width modulation
COP	Center of pressure
